# Evaluating practioners’ preferences regarding vascular emergency access in newborn infants in the delivery room: a national survey

**DOI:** 10.1186/s12887-020-02294-4

**Published:** 2020-08-27

**Authors:** Bianca Haase, Laila Springer, Christian Friedrich Poets

**Affiliations:** Department of Neonatology, University Children’s Hospital Tuebingen, Calwerstraße 7, 72076 Tuebingen, Germany

**Keywords:** Delivery room, Resuscitation, UVC placement, Intraosseous access, Venous access

## Abstract

**Background:**

Venous access during neonatal emergencies in the delivery room (DR) can be accomplished through an umbilical venous catheter (UVC) or an intraosseous (IO) access. Preference of one over the other is unclear. We wanted to evaluate practioners’ views.

**Methods:**

An anonymous online questionnaire was circulated to healthcare professionals with different background and experience, all working in neonatal intensive care units in Germany. The web-based survey consisted of 13 questions and data collection was performed using an online tool.

**Results:**

We received 502 completed questionnaires, 152 (30%) were from neonatologists, the remainder from residents, fellows and neonatal nurses. For resuscitation of term newborns in the DR 61% of neonatologists vs. 53% of non-neonatologists were in favour of UVC instead of an IO as an emergency access. UVC placement was rated (very) difficult to impossible by 60% of neonatologists and 90% of non-neonatologists (p < 0.05). All respondents cited lack of experience as the main reason for feeling reluctant to place an UVC or IO access, the latter only being taken into consideration in term infants.

**Conclusions:**

UVC placement in the DR is rated more often difficult to use by non-neonatologists than by neonatologists, apparently related to lack of experience. IO access was only considered for resuscitating term infants due to lacking practice and missing approval for birth weights < 3000 g. Frequent training might improve these clinical skills.

## Background

In the crucial first postnatal minutes the establishment of a venous access is essential especially in very premature infants and term newborns with circulatory compromise. This, however, may be challenging and time consuming [1] and untreated arterial hypotension or persistent bradycardia may ensue [2]. The 2015 ERC (European Resuscitation Council) guidelines for newborn resuscitation recommend an umbilical venous catheter for the administration of drugs (UVC) [3]. However, placing an UVC might be challenging and takes longer than an intraosseous (IO) access especially for untrained personnel [4]. Moreover, UVC placement as an invasive procedure entails additional risks, e.g. thrombosis [5] and necrotizing enterocolitis (NEC) [6]. If an intravenous vascular access is unsuccessful, the IO access seem to be a good alternative during resuscitation of critically ill neonates in comparison to the more sophisticated UVC placement procedure, especially for untrained personnel [7].

Therefore, IO cannulas should be available in all neonatal units and their application should be trained [8]. It is a non-natural access pathway, however, with a complication rate of 13% [9].

In order to verify whether prevailing practice during DR management corresponds to current guidelines, we developed a national online survey for healthcare professionals with different background and experience with a focus on the most commonly used access routes in a neonatal emergency setting.

## Methods

### Study design and consent

An anonymous web-based online survey was created using SurveyMonkey (San Mateo, USA) and circulated between 11/2018 and 1/2019 after approval by the Ethics Committee of Tuebingen University Hospital (871/2018). Since this is an anonymous data analysis of the SurveyMonkey platform, consent was given by voluntarily participation the questionnaire.

### Data collection

Data were collected using a web-based survey (Survey Monkey, San Mateo, USA), which was distributed among healthcare professionals via e-mail. While responses were anonymous, participants were asked to use an online link to receive a unique token to complete the survey, which was announced at neonatal workshops organised by the authors’ institution with the request to distribute the link among colleagues. There was no financial incentive for taking part in the survey.

#### Questionnaire

The questionnaire consisted of 13 questions (in German) and had been validated by 10 independent physicians and 10 nurses with regard to its comprehensibility, suitability of its pre-formulated answers, and simplicity. It differentiated between routine and emergency situations during DR management. Both, UVC and IO access, were evaluated for an emergency situation in the DR; the placement of an UVC was also evaluated in a non-emergency setting in non-depressed preterm infants. Non-emergency situations are routine situations in the DR with the need of a central line for administration of glucose or medications such as caffeine in extremely preterm infants. There was no evaluation for out-of-hospital use. The questionnaire contained single choice and multiple-choice responses; for 13 questions, an option to select “others” or a free-text field was also offered (Table 1). Questions could be answered with “very easy,” “easy”, difficult”, “very difficult” or “impossible”, but ratings were subsequently collapsed into “(very) easy” and “(very) difficult”. In order to standardize terminology, we adapted the German Level I-III system for neonatal care to the American classification. Centres were divided into tertiary-level neonatal intensive care units (NICUs) Level III centres (in Germany called ‘Level I centre’) and non-tertiary special care nurseries (SCNs) (in Germany classified as ‘Level II and Level III units’; Table 1).

**Table 1:** Questions of the online survey

**1.**	**PERSONAL DATA**		
**1.1**	**Professional group you belong to (SC)**		
a	neonatal nurses (non-neonatologists)	b	residents (non-neonatologists)
c	fellows (non-neonatologists)	d	senior physicians (neonatologists)
e	head of the neonatal department (neonatologist)		
**1.2**	**Your professional experience in years (SC)**		
a	0–3 years	b	3–7 years
c	more than 7 years	d	others (please specify)
**1.3**	**Your hospital’s NICU level of care (based on the German G-BA nomenclature) (SC)**		
a	Level I (equivalent to tertiary unites); admitting all infants		
b	Level II; admitting infants with a birthweight of > 1250 g or > 29 wk gestation		
c	Level III (equivalent to international NICU level I); admitting infants > 1500 g or > 32 wk gestation		
**1.4**	**Number of deliveries per year in your hospital (SC)**		
a	< 1000	b	1000–2000
c	> 2000	d	others (please specify)
**2.**	**CONVENTIONAL PLACEMENT OF AN UVC**		
**2.1**	**How many UVC have you placed successfully? (SC)**		
a	0	b	1–5
c	more than 5		
**2.2**	**On a scale of 1–5, how do you rate the feasibility of UVC insertion in a routine non-emergency setting in the DR? (SC)**		
a	very simple	b	simple
c	difficult	d	very difficult
e	impossible		
**2.3**	**On a scale of 1–5, how do you rate the feasibility of UVC insertion in an emergency setting in the delivery room? (SC)**		
a	very simple	b	simple
c	difficult	d	very difficult
e	impossible		
**2.4**	**What do you think are the most common problems during an UVC placement? (MC)?**		
a	time delay	b	catheter malposition
c	manpower (human resources)	d	lack of experience
e	others (please specify)		
**3.**	**INTRAOSSEOUS CANNULA (IOC) (SC)**		
**3.1**	**How many IOC have you performed successfully?**		
a	0	b	1–5
c	more than 5		
**3.2**	**On a scale of 1–5, how would you rate the feasibility of inserting an IOC in an emergency situation in the DR? (SC)**		
a	very simple	b	simple
c	difficult	d	very difficult
e	impossible		
**3.3**	**What do you think is(are) the most common problem(s) during IOC insertion? (MC)**		
a	causing Pain	b	potential for bone injury
c	extravasate	d	malposition
e	lack of experience	f	others (please specify)
**4.**	**PREVAILING PRACTICE**		
**4.1**	**In an emergency situation in the delivery room, which access route would you consider for a newborn weighing 4000 g (with pronounced centralisation)? (SC)**		
a	UVC	b	IOC
**4.2**	**For the initial delivery room treatment of a 500 g premature baby, which access route would you prefer (after failed placement of a peripheral venous line)? (SC)**		
a	UVC	b	IOC

Answers possible as *MC* Multiple Choice, *SC* Single Choice and free answering fields if named: Others (please specify)

#### Data analysis

Responses were imported from the SurveyMonkey database to SPSS version 25 (IBM, Chicago, IL). Descriptive statistics were generated for key variables including educational degrees of healthcare professionals, NICU level and annual number of deliveries. Categorical data were summarized and shown as counts and percentages. Ordinal data were analysed using Mann-Whitney U-test. A *p*-value (two-sided) of < 0.05 was considered to represent statistical significance.

## Results

We received 502 completed questionnaires, including 152 (30%) from neonatologists (Table 2). 395 respondents (79%) worked in tertiary-level NICUs, the remainder in non-tertiary SCNs. Approximately one half (54%) worked in hospitals with ≥ 2000 deliveries per year. 321 respondents (64%) indicated that they had at least three years of work experience in neonatology.

**Table 2:** Respondents of the online survey

**Survey consisting of 13 questions; respondents 502**	
**Neonatologists**	***n* = 152 (30.3%)**
**Non-neonatologists consisting of**:	***n* = 350 (69.7%)**
Residents	***n*** = 145 (28.9%)
Fellows	***n*** = 99 (19.7%)
Neonatal nurses	***n*** = 106 (21.1%)
Respondents working in Level III centres (German called NICU level I)	***n ***= 395 (78.8%)
Respondents working in Level II centres (German NICU level II)	***n*** = 60 (12%)
Respondents working in Level I centres (German NICU level III)	***n*** = 47 (9.4%)

Data are displayed as counts and percentages. *NICU* Neonatal Intensive Care Unit

50% of respondents stated they had never applied an IOC by themselves and 30% had no previous experience in establishing an UVC access.

In agreement with the above guidelines, for DR management 61% of neonatologists vs. 53% of non-neonatologists were in favour of UVC placement instead of an IO access for the resuscitation of a term newborn. In tertiary-level NICUs vs. non-tertiary SCNs, 57% vs. 50%, respectively, of respondents preferred an UVC placement in the DR.

**Fig. 1:**
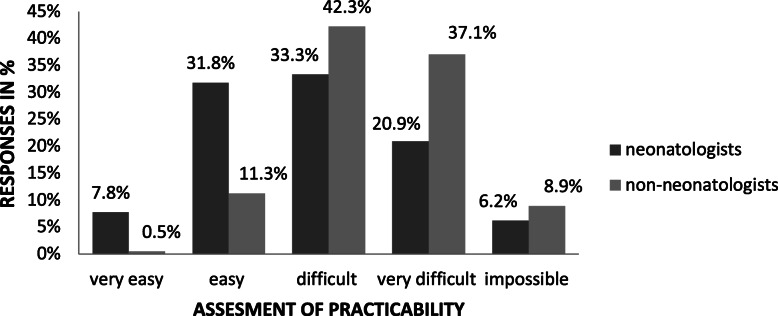
Compares the opinion of neonatologists respectively non-neonatologists of the practicability of an UVC in an emergency setting in the delivery room (*p* < 0.05)

While evaluating emergency UVC placement in the DR, almost 90% of non-neonatologists or respondents working in non-tertiary SCNs rated the procedure as (very) difficult to impossible, and even 60% of experienced neonatologists or respondents from tertiary-level NICUs considered it (very) difficult to impossible (*p* < 0.05) (Fig. 1). In all responses, lack of experience was cited as the main reason for a reluctance to place an UVC (53%).

**Fig. 2:**
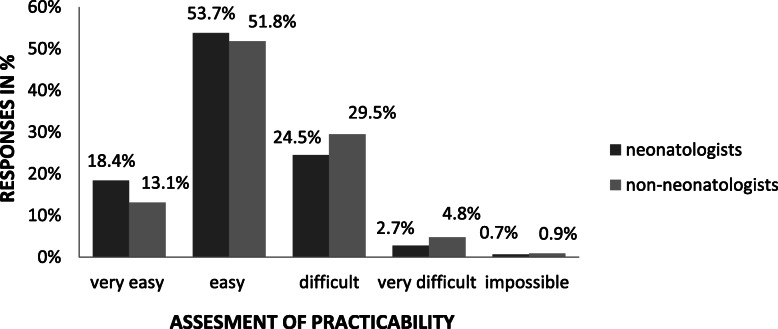
Compares the opinion of neonatologists respectively non-neonatologists on the difficulty in placing an intraosseous access in an emergency setting in the delivery room (*p* > 0.05). (cave: weight > 3000 g). There was no comparison between routine and emergency setting

Emergency application of an IO access (Fig. 2) in the DR was rated (very) easy by 72% of neonatologists vs. 65% of non-neonatologists, although 50% had no previous real-life experience with it, with this proportion being similar in tertiary-level NICUs versus non-tertiary SCNs. Reasons given for preference of an UVC over IOC included avoidance of pain (24%), a potential for bone injury (32%), catheter malposition (40%) or lack of experience (56%).

**Fig. 3:**
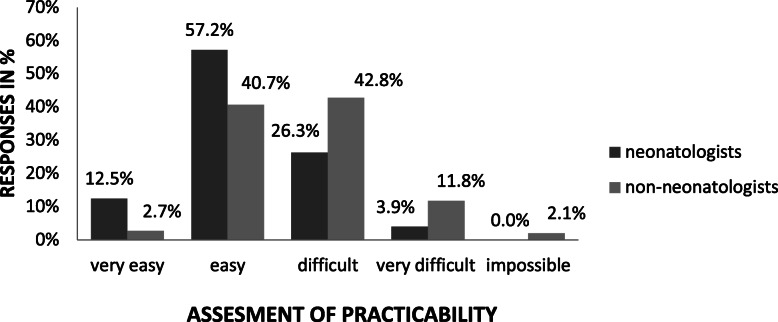
Compares the opinion of neonatologists respectively non-neonatologists on the difficulty in placing a UVC in the delivery room in a non-emergency setting in non-depressed preterm infants (*p* < 0.05)

In a non-emergency setting in the DR, UVC placement was evaluated to obtain a basic assessment of this procedure. 70% of responding neonatologists respectively 66% of respondents from tertiary centres rated the application of routine UVC placement as (very) easy, whereas only 43% of non-neonatologists (*p* < 0.05; Fig. 3), respectively 32% of non-tertiary centres, rated it as very easy (data not shown).

## Discussion

To our knowledge, this is the first national survey evaluating current opinions of healthcare professionals in Germany regarding placement of an UVC or IO access in an emergency setting in the DR. In accordance with current guidelines, responders preferred an UVC over IO access during transition at birth.

Only a narrow majority of 60% was in favour of an UVC in emergency situations in the DR, which could be due to the fact that establishing an IO access was classified as (very) easy by 67% of respondents, even though only 50% had ever implemented one themselves.

Respondents to our survey rated the level of difficulty according to their own level of training and experience, which might be a reason why, contrary to current recommendations, with less experience the affinity to IO access increased. While many extremely preterm infants born in tertiary centres need a central venous line access during their subsequent neonatal intensive care [10], the UVC is often placed either in the delivery room or shortly afterwards in the NICU [11], as it provides a painless and reliable vascular access for preterm infants avoiding the skin punctures needed for other forms of vascular access [12].

However, as long as the UVC remains the recommend access in DR management in international guidelines [3] and as long as there is a lack of a device that simplifies the inserting procedure, consistent training should be enforced [4].

An umbilical cord simulator may offer a realistic training with real human cords [13] and should be preferred to manikins with an artificial and more unrealistic umbilical cord [14].

Another reason for placing an UVC is that high plasma levels of epinephrine can be reached faster and more reliably via a centrally positioned UVC than via the endotracheal route [15] and, according to the 2015 ERC Guidelines, drugs should be applied this way [3]. It remains unclear whether the same is true for an IO access [16]. Initial studies (excluding neonatal patients) showed no significant interaction between the access route and study drug outcomes [16]. However, IO access in neonates has not yet been investigated in detail, only case series, post-mortem studies and simulation studies could be identified and showed a lack of evidence in this patient group [8].

However, successful placement of an UVC took 46 s longer than application of an IO access in a simulation study [17]. In an emergency situation in the DR, this delay may be responsible for the increasing preference of an IO access during the resuscitation of term neonates, as confirmed by our survey. Therefore, such IO access should be available, trained and taken into consideration on all neonatal units if other access routes have failed [18].

Previous experience with IO access significantly reduced reluctance and increased the willingness to use an IO access as the first choice for emergency vascular access [19].

Besides that, the IO access is a non-natural access route with a complication rate of 13% [9] such as fractures, limb ischaemia and need for amputation [20, 21]. The complications are higher in smaller infants due to the small margin of error when inserting an IO device [22].The “risk of a bone injury” and “causing pain” were the main reasons cited in our survey why respondents would not apply an IO access.

A recent trial in 16 stillborns showed success rates in newborns between 40% and 60%, depending on the needle used [22]. However, checking the correct position of an IO access with a CT-scan is difficult to accomplish during neonatal resuscitation.

Moreover, a major problem of UVC placement is malposition, which is associated with a higher risk of thrombosis [5] and NEC [6]. In our survey, a risk of malposition was mentioned 72 times in the free-text option when respondents were asked to mention most common problems during UVC insertion.

This leads to the first limitation of our study: only few multiple-choice responses were used, thus “malposition” was not offered as a response. Furthermore, we did not compare blunt hollow cannulas with umbilical venous catheters, this might have been interesting especially in the context of resuscitation in the DR.

Additionally, we included fewer open questions to minimize the risk of having a low participation rate.

Another limitation is that distribution of the questionnaire was random and therefore not all neonatal centres might have been reached. This was due to the fact that there is no general email list for specified healthcare professionals in neonatology, which may have introduced a selection bias. In addition to that, GDPR (General Data Protection Regulation) rules dictated anonymity of the e-mail responses received, prohibiting us from gathering data on the number of participants and refusals to participation.

Nevertheless, a strength of our study is a high number of respondents during the 3-month period so that we can still present a broad picture of opinions.

## Conclusions

UVC placement in an emergency setting in the DR was rated more difficult by non-neonatologists compared to neonatologists in this German online survey, mainly due to the perceived difficulties in performing an UVC placement and lack of experience; both of which can only be improved by frequent training until there is a device that simplifies the sophisticated and challenging process of placing a UVC.

For this reason, inserting an IO access, which is much easier to accomplish, may continue to be justified during resuscitation of term neonates and should be trained and available in all neonatal units.

## Data Availability

The datasets used and/or analysed during the current study are available from the corresponding author on reasonable request.

## Abbreviations

CT-scan: Computer Tomography Scan
DR: Delivery room
ERC: European Resuscitation Council
GDPR: General Data Protection Regulation
ILCOR: International Liaison Committee on Resuscitation
IO: Intraosseous
NEC: Necrotizing enterocolitis
NICU: Neonatal intensive care unit
SCN: Special Care Nursery
UVC: Umbilical venous catheter

## References

[1] Atkins DL, de Caen AR, Berger S, Samson RA, Schexnayder SM, Joyner BL (2018). 2017 American Heart Association focused update on pediatric basic life support and cardiopulmonary resuscitation quality: an update to the American Heart Association guidelines for cardiopulmonary resuscitation and emergency cardiovascular care. Circulation.

[2] Rong Z, Liu H, Xia S, Chang L (2012). Risk and protective factors of intraventricular hemorrhage in preterm babies in Wuhan, China. Childs Nerv Syst.

[3] Wyllie J, Bruinenberg J, Roehr CC, Rudiger M, Trevisanuto D, Urlesberger B (2015). European Resuscitation Council Guidelines for Resuscitation 2015: Sect. 7. Resuscitation and support of transition of babies at birth. Resuscitation.

[4] Schwindt EM, Hoffmann F, Deindl P, Waldhoer TJ, Schwindt JC (2018). Duration to Establish an Emergency Vascular Access and How to Accelerate It: A Simulation-Based Study Performed in Real-Life Neonatal Resuscitation Rooms. Pediatr Crit Care Med.

[5] Sherwani P, Vire A, Anand R, Jajoo M (2016). Umbilical venous catheterization gone wrong: Hepatic complications. Indian J Radiol Imaging.

[6] Sulemanji M, Vakili K, Zurakowski D, Tworetzky W, Fishman SJ, Kim HB (2017). Umbilical Venous Catheter Malposition Is Associated with Necrotizing Enterocolitis in Premature Infants. Neonatology.

[7] Perlman JM, Wyllie J, Kattwinkel J, Wyckoff MH, Aziz K, Guinsburg R (2015). Part 7: Neonatal Resuscitation: 2015 International Consensus on Cardiopulmonary Resuscitation and Emergency Cardiovascular Care Science With Treatment Recommendations (Reprint). Pediatrics.

[8] Scrivens A, Reynolds PR, Emery FE, Roberts CT, Polglase GR, Hooper SB (2019). Use of Intraosseous Needles in Neonates: A Systematic Review. Neonatology.

[9] Ellemunter H, Simma B, Trawoger R, Maurer H (1999). Intraosseous lines in preterm and full term neonates. Arch Dis Child Fetal Neonatal Ed.

[10] Sawyer T, French H, Ades A, Johnston L (2016). Neonatal-perinatal medicine fellow procedural experience and competency determination: results of a national survey. J Perinatol.

[11] Meaney PA, Bobrow BJ, Mancini ME, Christenson J, de Caen AR, Bhanji F (2013). Cardiopulmonary resuscitation quality: [corrected] improving cardiac resuscitation outcomes both inside and outside the hospital: a consensus statement from the American Heart Association. Circulation.

[12] Sanderson E, Yeo KT, Wang AY, Callander I, Bajuk B, Bolisetty S (2017). Dwell time and risk of central-line-associated bloodstream infection in neonates. J Hosp Infect.

[13] Sawyer T, Gray M, Hendrickson M, Jacobson E, Umoren R (2018). A Real Human Umbilical Cord Simulator Model for Emergency Umbilical Venous Catheter Placement Training. Cureus.

[14] Sawyer T, Starr M, Jones M, Hendrickson M, Bosque E, McPhillips H (2017). Real vs simulated umbilical cords for emergency umbilical catheterization training: a randomized crossover study. J Perinatol.

[15] Vali P, Chandrasekharan P, Rawat M, Gugino S, Koenigsknecht C, Helman J (2017). Evaluation of Timing and Route of Epinephrine in a Neonatal Model of Asphyxial Arrest. J Am Heart Assoc..

[16] Granfeldt A, Avis SR, Lind PC, Holmberg MJ, Kleinman M, Maconochie I (2020). Intravenous vs. intraosseous administration of drugs during cardiac arrest: A systematic review. Resuscitation.

[17] Rajani AK, Chitkara R, Oehlert J, Halamek LP (2011). Comparison of umbilical venous and intraosseous access during simulated neonatal resuscitation. Pediatrics.

[18] Scrivens A, Reynolds PR, Emery FE, Roberts CT, Polglase GR, Hooper SB, et al. Use of Intraosseous Needles in Neonates: A Systematic Review. Neonatology. 2019;116(4):305-14. https://www.ncbi.nlm.nih.gov/pubmed/31658465. 10.1159/000502212 31658465

[19] Lo TY, Reynolds F (2009). To use intraosseous access or not to use intraosseous access: determinants of trainees’ decision in paediatric emergencies. Eur J Emerg Med.

[20] Suominen PK, Nurmi E, Lauerma K (2015). Intraosseous access in neonates and infants: risk of severe complications - a case report. Acta Anaesthesiol Scand.

[21] Katz DS, Wojtowycz AR (1994). Tibial fracture: a complication of intraosseous infusion. Am J Emerg Med.

[22] Fuchs Z, Scaal M, Haverkamp H, Koerber F, Persigehl T, Eifinger F (2018). Anatomical investigations on intraosseous access in stillborns - Comparison of different devices and techniques. Resuscitation.

